# Arousal—But Not Valence—Reduces False Memories at Retrieval

**DOI:** 10.1371/journal.pone.0148716

**Published:** 2016-03-03

**Authors:** Chiara Mirandola, Enrico Toffalini

**Affiliations:** Department of General Psychology, University of Padova, via Venezia 8, 35131 Padova, Italy; Technion—Israel Institute of Technology, ISRAEL

## Abstract

Mood affects both memory accuracy and memory distortions. However, some aspects of this relation are still poorly understood: (1) whether valence and arousal equally affect false memory production, and (2) whether retrieval-related processes matter; the extant literature typically shows that mood influences memory performance when it is induced before encoding, leaving unsolved whether mood induced before retrieval also impacts memory. We examined how negative, positive, and neutral mood induced before retrieval affected inferential false memories and related subjective memory experiences. A recognition-memory paradigm for photographs depicting script-like events was employed. Results showed that individuals in both negative and positive moods–similar in arousal levels–correctly recognized more target events and endorsed fewer false memories (and these errors were linked to *remember* responses less frequently), compared to individuals in neutral mood. This suggests that arousal (but not valence) predicted memory performance; furthermore, we found that arousal ratings provided by participants were more adequate predictors of memory performance than their actual belonging to either positive, negative or neutral mood groups. These findings suggest that arousal has a primary role in affecting memory, and that mood exerts its power on true and false memory even when induced at retrieval.

## Introduction

Memory illusions have been in the spotlight of cognitive and neurocognitive scientists for decades. The discovery that memory does not lead to static representations of events is now a milestone concept; memory scientists have begun to investigate in detail the numerous variables that influence the occurrence of distortions, including the emotional state or mood of the rememberer. The present study is focused on the effect of transient mood (negative, positive, and neutral), induced immediately before retrieval, on the creation of memory distortions for everyday scripted events.

When individuals are exposed to mood-inducing emotional stimuli, the tendency to incur memory errors—or false memories—may depend on the valence of their affective state. Some studies have shown that negative mood reduces and positive mood enhances memory errors [1–3], and that this is especially true when mood is induced before learning. In these studies, the Deese-Roediger-McDermott paradigm (DRM; [4]) was employed. The DRM paradigm consists of the presentation of a series of wordlists including semantically related words (e.g., *nurse*, *sick*, *medicine*). Within each list, words also converge in meaning toward a non-presented lure (e.g., *doctor*); the acceptance of this word at recognition represents a false memory. According to the affect-as-information hypothesis, negative mood affects encoding as it favors item-specific processing, which limits the incorporation of non-presented lures; on the other hand, positive mood promotes relational processing, which facilitates the endorsement of items that share the semantic properties of the encoded events [5].

Other studies using the same paradigm have found that arousal (i.e., level of activation) induced before encoding, and not valence per se, affects the likelihood to incur memory errors. Nonetheless, evidence is mixed: in some cases, high arousal has been found to elicit higher false recall and false recognition than low arousal [6], but also the opposite has been found, with high arousal leading to fewer memory errors than low arousal [7]. Importantly, arousal exerts its influence on memory distortions not only at encoding but also when induced after learning: indeed, it has been found to reduce acceptance of misleading information in a misinformation paradigm [8]. The authors of this study argue that arousal enhanced memory consolidation via modulation of the hippocampal memory system by the amygdala; indeed, the amygdala is activated by many different emotional conditions [9]. However, in this study only negative-arousing and neutral stimuli were employed to induce mood, making it difficult to draw conclusions about the influence of valence on false memory production.

Furthermore, a number of studies have shown that high levels of stress influence subsequent true and false memory. For example, stress has been found to reduce false memories when induced either before encoding [10] or before retrieval [11]. However, other studies found that stress experienced at retrieval did not affect false memories, but only hindered true memory [12], and that stress experienced prior to learning DRM wordlists enhanced subsequent critical lures endorsement [13]. While deriving different conclusions, all these studies highlight the importance of the biological mechanisms underlying stress/arousal influence on memory; stress hormones (i.e., cortisol and adrenaline/noradrenaline) influence hippocampal activity, and through interaction with the amygdala they can mediate learning and subsequent memory, especially for emotional events. All these studies focused on induced stress leading to negative and disturbing perceived arousal; they were not aimed at studying the role of arousing pleasant events, leaving unsolved what the different impact of both valence and arousal on memory would be.

Whether valence or arousal play a major role and whether retrieval-related processes are important in modulating the influence of mood on memory is still poorly understood. Bless and colleagues [14] did not find evidence of a distinct influence of mood on false memories when induced at encoding or retrieval. In their study, participants had to listen to a script-based story, on which they were later going to be tested; either before or after encoding the story, they were asked to provide a written report of a personal life event which had to be either happy (inducing a happy mood) or sad (inducing a sad mood). The authors found that–regardless of time of mood induction–people in happy moods were more likely than people in sad moods to incorporate non-presented typical information into their memory, given their higher reliance on the general knowledge structure resulting from the script. However, as in Storbeck and Clore’s study [3], the emotional negative condition was low-arousing; thus, whether high arousing stimuli presented before retrieval influence subsequent true and false memories is still to be clarified.

The present study ought to clarify the effect of induced mood on false memories for everyday events, comparing high-arousal mood states of positive and negative valence with low-arousal mood of neutral valence, concurrently analyzing objective and subjective measures of false recognition. A false-memory paradigm for photographs depicting script-like events [15] was employed; this paradigm allows for the investigation of two types of errors which arise when an individual remembers either a script-consistent but non-presented event (i.e., gap-filling error) or the non-presented cause of a studied action effect (i.e., inferential causal error [16]; for further adaptations see [17–20]). For example, in the “family dinner” script, a gap-filling error could result from wrongly recognizing the adult protagonists eating pasta, whereas instead they were drinking wine; and a causal error could be caused by wrongly recognizing the cause of an action such as the child protagonist knocking over a bottle of water, whereas only the effect scene had actually been presented (e.g., pieces of a broken bottle on the floor). Furthermore, subjective measures of recollection and familiarity were collected.

The assessment of the influence of mood on subjective experiences related to memory errors has been neglected thus far. The remember-know paradigm [21] requires introspection about one’s own memory states in order to provide a more detailed recognition response at the time of testing. Remember judgments are to be given not only when the person is able to recognize a certain item as previously seen, among new ones, but also when some qualitative features are available, including personal thoughts or feelings that were linked to the encoding of the item. Know judgments reflect, instead, that the person is able to recognize a certain item, but no other qualitative features are available.

Recently, one study showed that participants in a negative mood associated more remember responses to negative DRM critical lures compared to neutral critical lures, and this happened both at immediate recognition and one week later [22]. However, a positive mood group was not included in this study, thereby preventing the specific assessment of differently valenced emotional states. Another study [23] found mood-congruent subjective recollection for false remembering—that is, individuals in negative moods associated more remember responses to negative DRM wordlists than either positive or neutral mood groups, and individuals in positive moods associated more remember responses to positive wordlists compared to negative and neutral. In the current study, asking participants to provide remember-know judgments has a twofold objective: on the one hand it would provide qualitative information on the nature of memory errors; on the other hand, it would help clarifying the importance of retrieval-related processes (such as monitoring strategies based on recollection).

In the present study, mood was induced immediately before recognition, in order to study retrieval-related processes. Based on extant studies which investigated the influence of valenced mood—induced before retrieval—on false memories [3, 14], one could expect that positive and negative mood states would influence false memory production to a different extent (with negative mood preventing individuals from incurring false memories and positive mood enhancing the probability of committing errors). However, these studies only examined low arousal negative condition (i.e., sadness), making it difficult to evaluate the effect of arousal. Based on research showing that arousal induced after learning diminishes the propensity to incur distortions [8], one could expect that both positive and negative moods would prevent individuals from incurring memory errors compared with neutral mood. Most important, to our knowledge, the effect of arousing mood at retrieval on false memories for everyday events has not been studied yet. In the current study, positive and negative stimuli used to induce moods were equated for arousal levels, allowing for the investigation of the specific role of valence and arousal: if differences in false memory production should emerge between positive and negative moods, then valence plays a major role. However, if differences emerge between neutral mood and both positive and negative moods, then arousal must be considered as being the central factor that affects false memory formation.

Finally, as typically done in this field of research [e.g., 3; 7], the experiment was designed to test participants belonging to different mood groups. Therefore data analyses were initially conducted by comparing groups against each other. However, at a later stage of the analyses we wanted to investigate whether using the participants’ own ratings on their perceived valence and arousal levels before and after mood-induction would better predict memory outcomes compared to analyzing the impact of mood on false memory in a way that categorizes participants as belonging to either one of three mood groups (i.e., positive, negative or neutral). Indeed, while traditionally used in studies with mood induction, comparisons between groups neglect part of the variability, as they overlook the fact that within the same group one participant may be strongly affected by mood induction, while another may be only mildly affected.

## Materials and Methods

### Participants

A total of 75 Italian undergraduate students including 32 females and 43 males from University of Padova volunteered in this experiment (*M* = 22.8 years, *SD* = 1.8). Participants were exposed to either negative, positive or neutral mood (*n* = 25 for each group) through the presentation of a selection of pictures taken from the *International Affective Picture System* (IAPS; 24) that have been proven effective in inducing emotional states in previous research (e.g., [1, 25]). The study met the ethical requirements established for research by the Italian Psychological Association (AIP). The study has been approved by the Ethical Committee for Psychological Research (Area 17) of University of Padova. Participants were informed of the study’s general goals and selected on the basis of preliminary answers to a brief survey investigating fears or phobias related to wounds, blood, and so forth. Participants with specific phobias were excluded from participation in the study. Written informed consent was obtained by all participants prior to task administration.

### Mood-induction

A selection of pictures was derived from the IAPS [24] standardized ratings, which range from 1 (negative valence or low arousal) to 9 (positive valence or high arousal). Twenty-four pictures were included in each of the three mood conditions, following specific criteria: negative pictures had low valence (*M* = 2.03, *SD* = .34) and high arousal (*M* = 6.81, *SD* = .35); positive pictures had high valence (*M* = 7.07, *SD* = .42) and high arousal (*M* = 6.53, *SD* = .32); neutral pictures had average valence (*M* = 5.02, *SD* = .28) and low arousal (*M* = 2.87, *SD* = .52). Neutral pictures were purposely selected with low arousal, following Bradley and Lang’s recommendation [26] that pictures used to elicit a neutral condition should be typically characterized by moderate valence and low arousal. A one-way ANOVA revealed that valence significantly differed among the three groups, *F*(2,69) = 1233.97, *p* < .001, *η*_*p*_^*2*^ = .97; post-hoc tests (with Bonferroni correction) showed that negative pictures were characterized by lower valence than both positive and neutral, and positive pictures were characterized by higher valence than both negative and neutral pictures. Further, a one-way ANOVA also showed that arousal differed among the three groups, *F*(2,69) = 691.05, *p* < .001, *η*_*p*_^*2*^ = .95. Post-hoc tests (with Bonferroni correction) showed that negative and positive pictures (which did not differ between each other) had higher arousal than neutral pictures.

### Self-assessment manikin

SAM scales [27] were used both prior to the experiment (pre-SAM) and after the presentation of the IAPS pictures (post-SAM) to ensure that mood-induction was effective. Each SAM scale consisted of a 9-point rating scale and was used by the participants to rate how they felt in terms of happiness/unhappiness (valence) and activation/non-activation (arousal); nine refers to both high arousal and high valence, and 1 to low arousal and low valence. This rating scale has been used in previous work to assess the effectiveness of mood induction (e.g., [22]).

### False memory paradigm

#### Encoding

Color photographs depicting individuals engaged in 8 everyday episodes, or scripts, were presented. The episodes included: having a family dinner at home, playing at the playground, going shopping, waking up, doing homework after school, going on a bike trip, going to the doctor, and children performing a drama piece in a theater (see [15], for an example of the family dinner episode). We note that the protagonists of the episodes were children because the paradigm has been designed to be suitable for developmental studies (such as in [17]). For each episode, 11 photographs depicted the typical actions for that event; embedded in these photographs was one photograph representing the effect of an action, whose corresponding cause was not presented. Finally, 10 photographs inconsistent with any of the eight episodes were presented at the beginning and at the end of the presentation in order to avoid possible primacy and recency effects on the relevant material. These photographs included children doing different activities such as playing an instrument, going to a party, and so forth.

#### Retrieval

A unique sequence of 80 photographs was employed. For each of the eight episodes, the recognition test included: (a) four target photographs—that is, photographs presented at encoding; (b) three new photographs consistent with one of the scripts but not presented at encoding—for the waking up script, the new photograph might depict a girl brushing her teeth whereas she was combing her hair in the picture presented at encoding; (c) one cause photograph whose effect had been presented during the encoding phase, such as a photo of a child knocking over a glass bottle to correspond to the effect scene depicting pieces of a broken bottle on the floor and a father about to hit his son; (d) one target photograph presented at encoding that was inconsistent with any of the scripts; and (e) one new, non-presented photograph inconsistent with any of the scripts.

### Procedure

All participants were tested individually at the Department of General Psychology, University of Padova. After 5 minutes of general conversation, participants were invited to indicate how they felt in that particular moment on both the dimensions of valence and arousal, using the 9-point pre-SAM scales. They were then given instructions for the encoding phase of the false memory task; specifically, they were told that they would see photographs depicting everyday situations and that they would have to pay close attention, trying to understand the story they represented. Participants were not told that they were later going to be tested on memory for those events. Photographs of the eight episodes were then presented in a logical and temporal order, appearing on the computer screen for 2 seconds, followed by a 2-second black slide. The duration of encoding phase was approximately 7 minutes.

After receiving the instructions for the encoding phase, participants were immediately administered the false memory paradigm. As soon as the encoding phase ended, a 15-minute retention interval followed, during which participants were first administered filler tasks followed by the mood-induction procedure (which took place immediately before retrieval). Instructions for the retrieval phase were given before the mood-induction procedure, in order to ensure that mood would exert its influence on retrieval. The IAPS pictures were presented on the computer screen for 5 seconds each. Following the mood induction, all participants were required to complete the post-SAM ratings.

For the recognition test, all participants were told that they would be presented with old and new photographs and that for each photograph they were to respond with “yes” if they saw it at encoding or “no” if they did not. In order to evaluate the subjective memory experiences, remember-familiar judgments were required for each recognized item. “Familiar” was used instead of “know” because it is easier to understand, especially in Italian [28]. Participants were instructed to select the option “remember” when they could clearly remember that picture and could further remember some qualitative features related to its encounter, such as a specific object in the scene or some thought that came to mind when they first viewed it. They were asked to select “familiar” when they could recognize the picture (even with high confidence) but no other detail came to mind.

## Results

### Mood manipulation check

A mixed 3 (mood group: negative vs. positive vs. neutral) × 2 (pre-mood vs. post-mood induction) ANOVA, with mean valence ratings as the dependent measure, revealed a significant interaction between mood group and pre-post mood induction condition, *F*(2,72) = 27.12, *p* < .001, *η*_*p*_^*2*^ = .43; post hoc analyses (with Bonferroni correction) showed that valence decreased in the negative group (*p* < .001) and increased in the positive group (*p* = .04), while it did not change in the neutral group. In addition, groups did not differ in valence in pre-mood condition (all *p*s > .87), whereas they did differ in post-SAM reports, with valence being lower in the negative group than both the positive and neutral groups (*p*s < .001). A similar analysis conducted on the mean arousal ratings as the dependent measure revealed a significant interaction between mood group and pre- and post-mood induction, *F*(2,72) = 47.79, *p* < .001, *η*_*p*_^*2*^ = .57. Post hoc analyses (with Bonferroni correction) showed that arousal increased in both negative and positive mood groups (*p*s < .001), whereas it did not change in the neutral group (*p* = .26). Further, groups did not differ in arousal ratings prior to mood induction (all *p*s > .90), whereas they did differ in post-SAM reports: arousal was higher in both negative and positive groups than in the neutral group (all *p*s < .001), and it did not differ between negative and positive groups (*p* = .61). See Table 1 for the descriptive statistics.

**Table 1:** Mean valence and arousal ratings before (1) and after (2) mood induction.

	*Valence*		*Arousal*	
*Mood Group*	*SAM (1)*	*SAM (2)*	*SAM (1)*	*SAM (2)*
Negative	5.80 (1.66)	3.96 (1.84)	3.72 (1.86)	6.96 (1.69)
Positive	6.28 (1.57)	6.76 (1.45)	3.44 (1.83)	6.32 (1.84)
Neutral	6.24 (1.56)	6.12 (1.39)	3.80 (1.96)	3.48 (1.76)

*Note*. Standard deviations are reported in parentheses

### Memory performance

#### False recognition

To analyse the effect of mood on false recognition, the responses to causal and script-consistent distractor photographs were considered. As responses were of a binomial type (“yes”: 1, or “no”: 0), a logistic mixed-effects model approach was used [29, 30]. Causal and gap-filling errors were the “yes” responses to causal and new script-consistent photographs, respectively. In both cases, the fixed effect was Group (3 levels: negative, positive, neutral), while Participants and Scripts were treated as random effects. The significance of both fixed and random effects was tested through a series of likelihood ratio tests for nested models based on the chi-square distribution [31]. The Akaike Information Criterion (AIC; [32]) was also reported for each model; lower AIC indicates a better model. Odds ratios were used as a measure of effect size.

**Fig 1:**
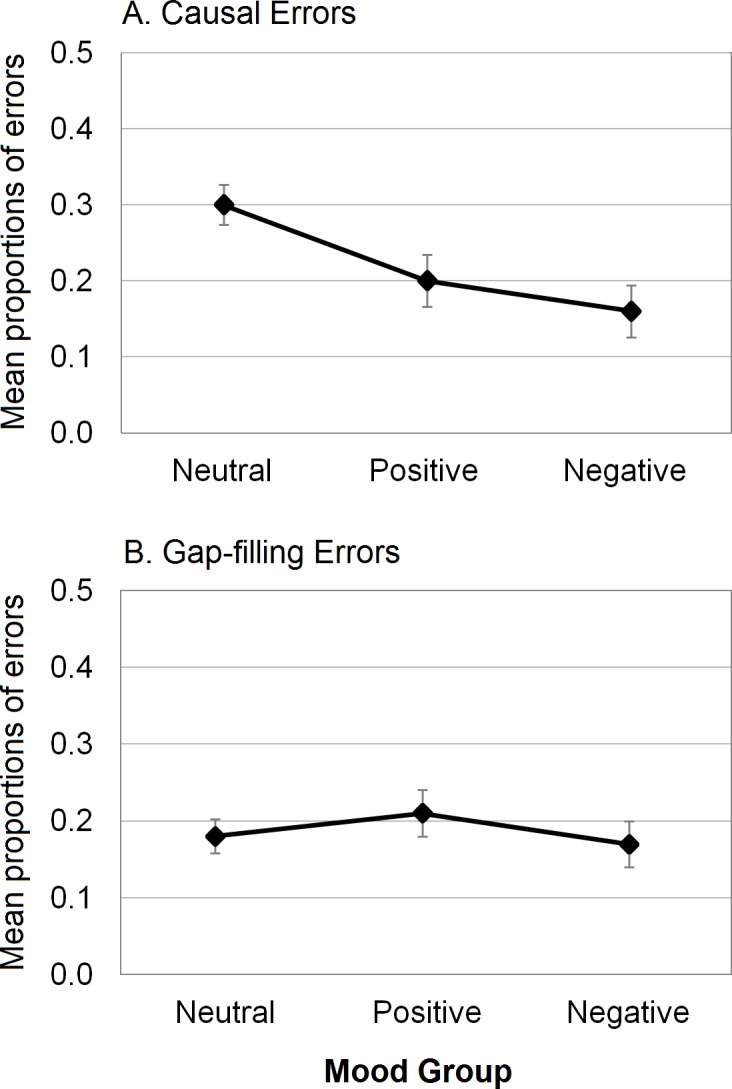
**Mean proportions of causal errors (A) and gap-filling errors (B) as a function of mood group**; error bars represent standard errors.

For causal errors, both random effects (i.e., participants and scripts) were significant: for Participants, *χ*^*2*^(1) = 4.89, *p* = .03 (full model: AIC = 543.3; model without Participants: AIC = 546.2); for Scripts, *χ*^*2*^(1) = 87.33, *p* < .001 (model without Scripts: AIC = 628.6). With regard to the fixed effect of Group, a significant main effect was found, *χ*^*2*^(2) = 9.75, *p* = .008 (model without Group: AIC = 549.1). The probability of producing causal errors in the three groups is reported in Fig 1A. Planned comparisons showed that the probability of producing causal errors was lower in both negative and positive groups than in the neutral group (*p*s < .05). See Table 2 for detailed information on the model, including estimated parameters and odds ratios.

**Table 2:** Fixed effect of Group on Causal Errors, Gap-filling Errors, and Hits using a logistic mixed-effects model.

*Fixed effect*	*B*	*SE*	*Odds ratio*	*χ*^*2*^*(df)*
*Dependent variable*: *Causal Errors*	* *	* *	* *	* *
Group				9.75** (2)
Positive Mood	- .65	.32	.52*	
Negative Mood	-1.04	.33	.35**	
*Dependent variable*: *Gap-filling Errors*	* *	* *	* *	* *
Group				.78 (2)
Positive Mood	.07	.35	1.07	
Negative Mood	-.23	.35	.79	
*Dependent variable*: *Hits*	* *	* *	* *	* *
Group				7.85* (2)
Positive Mood	.53	.27	1.70*	
Negative Mood	.77	.27	2.15*	

Baseline category for Group was "Neutral Mood". Random effects were Participants and Scripts. Number of observations was 2436 for Hits, 1764 for Gap-filling Errors, 600 for Causal Errors. Number of participants = 75. Number of scripts = 8.

*p < .05

**p < .01

For gap-filling errors, both random effects were significant: for Participants, *χ*^*2*^(1) = 102.22, *p* < .001 (full model: AIC = 1476.3; model without Participants: AIC = 1576.5); for Scripts, *χ*^*2*^(1) = 170.38, *p* < .001 (model without Scripts: AIC = 1644.7). The fixed effect of Group on gap-filling errors was not significant, *χ*^*2*^(2) = 0.78, *p* = .68 (model without Group: AIC = 1473.1). The probability of producing gap-filling errors in the three groups is reported in Fig 1B. See Table 2 for detailed information.

Responses to script-inconsistent distractors were not included in the analysis given that false alarms relative to these items approached floor effect (5%). They were included in the paradigm only to avoid primacy and recency effects on the relevant material.

#### Accuracy

**Fig 2:**
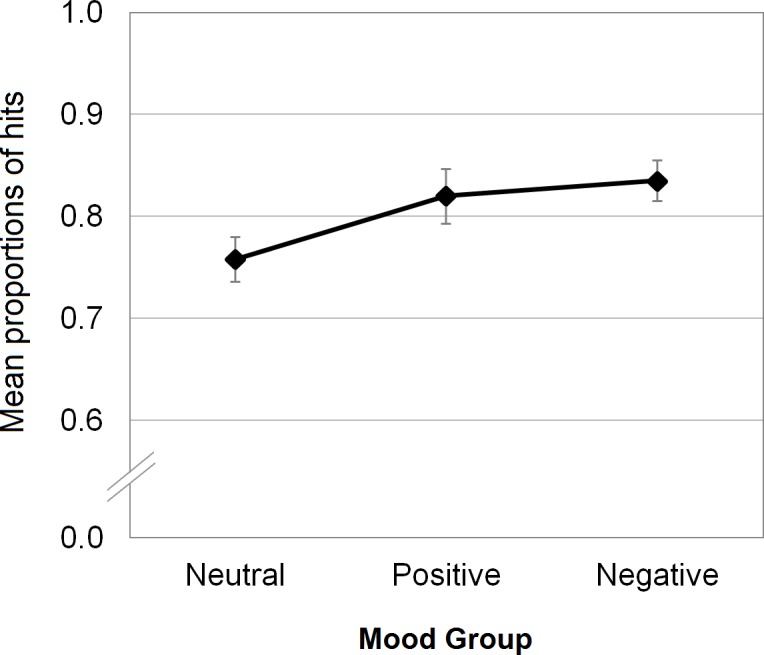
Mean proportions of hits as a function of mood group; error bars represent standard errors.

A similar logistic mixed-effects model was tested to analyse the effect of mood on hits (i.e., the “yes” responses to targets; “yes”: 1, “no”: 0). As above, the fixed effect was Group, and random effects were Participants and Scripts. Both random effects emerged as significant: for Participants, *χ*^*2*^(1) = 101.01, *p* < .001 (full model: AIC = 2143.4; model without Participants: AIC = 2242.3); for Scripts, *χ*^*2*^(1) = 180.76, *p* < .001 (model without Scripts: AIC = 2322.1). With regard to the fixed effect of Group, a significant main effect was found, *χ*^*2*^(2) = 7.85, *p* = .02 (model without Group: AIC = 2147.2). Planned comparisons showed that the probability of correctly recognizing target photographs was higher in both negative and positive groups than in the neutral group (negative: *p* = .005; positive: *p* = .05 see Fig 2). See Fig 2 and Table 2 for detailed information.

#### Remember and familiar responses

Additional analyses were performed to examine whether *remember* and *familiar* responses associated with causal errors and hits would change according to mood. Again, Participants and Scripts were treated as random effects, and significance was tested through likelihood ratio tests for nested models.

The analysis on the *remember* responses associated with causal errors revealed a significant main effect of Group, *χ*^*2*^(2) = 14.46, *p* < .001 (full model: AIC = 283.11; model without Group: AIC = 293.57). Planned comparisons showed that the probability of associating *remember* responses to causal errors was lower in both negative and positive groups than in the neutral group (*p*s < .05). On the contrary, with regard to *familiar* responses associated with causal errors, no main effect of Group was found, *χ*^*2*^(2) = 0.74, *p* = .69 (full model: AIC = 463.91; model without Group: AIC = 460.65). See Table 3 for detailed information on the models.

**Table 3:** Fixed effect of Group on *remember* and *familiar* responses associated with Causal Errors.

*Fixed effect*	*B*	*SE*	*Odds ratio*	*χ*^*2*^*(df)*
*Remember* responses	* *	* *	* *	* *
Group				14.46*** (2)
Positive Mood	-.95	.47	.39*	
Negative Mood	-2.11	.59	.12***	
*Familiar* responses	* *	* *	* *	* *
Group				.74 (2)
Positive Mood	-.22	.30	.46	
Negative Mood	-.22	.30	.46	

Baseline category for Group was "Neutral Mood". Random effects were Participants and Scripts. Number of observations was 600. Number of participants = 75. Number of scripts = 8.

*p < .05

***p < .001.

The analysis on the *remember* responses associated with hits did not show any significant main effect of Group, *χ*^*2*^(2) = 2.08, *p* = .35 (full model: AIC = 1891.7; model without Group: AIC = 1889.8). Furthermore, the analysis on the *familiar* responses associated with hits did not show any main effect of Group as well, *χ*^*2*^(2) = 2.24, *p* = .33 (full model: AIC = 1887.1; model without Group: AIC = 1885.3).

#### Comparing mood ratings vs. groups in predicting memory performance

To test whether actual mood ratings predict memory performance better than groups, we computed another series of logistic mixed-effects models on causal errors, gap-filling errors, and hits, but using the actual ratings reported by the participants through SAM as the predictors. In particular, we calculated differential valence and differential arousal as the difference between the valence and arousal ratings reported before and after mood induction. The significance of these predictors was tested through a series of likelihood ratio tests. Participants and Scripts were again included as random effects. Finally, the resulting models were compared with the corresponding models having group as the predictor. As the to-be-compared models are non-nested, comparisons were made through the relative likelihood based on the models’ AICs–this quantifies the strength of evidence in favour of one model over the other [33]; specifically, the relative likelihood was calculated as Exp((AIC_1_-AIC_2_)/2) [34].

For causal errors, differential valence did not have a significant effect, *χ*^*2*^(1) = 2.31, *p* = .13 (model with differential valence: AIC = 548.8; model without differential valence: AIC = 549.1), but a significant main effect of differential arousal was found, *χ*^*2*^(1) = 14.57, *p* < .001 (model with differential arousal: AIC = 536.5; model without differential arousal: AIC = 549.1); as shown by the parameters reported in Table 4, this indicates decreasing probability of causal errors with increasing arousal ratings. No interaction between differential arousal and valence emerged, *χ*^*2*^(1) = .57, *p* = .45 (model with the interaction: AIC = 539.0; model without interaction: AIC = 537.6). The relative likelihood of the model with differential arousal as the predictor (i.e. the best model) with respect to the corresponding model with group was 30.3, indicating clear evidence in favour of differential arousal (following [33], this implies that the first of the two models is 30.3 times more likely to be the best model than the latter).

**Table 4:** Fixed effect of differential valence and differential arousal on Causal Errors, Gap-filling Errors, and Hits using logistic mixed-effects models.

*Fixed effect*	*B*	*SE*	*Odds ratio*	*χ*^*2*^*(df)*
*Dependent variable*: *Causal Errors*				
Differential valence	.15	.10	1.16	2.31 (1)
Differential arousal	- .25	.07	.78***	14.57*** (1)
Differential valence x Differential arousal	- .04	.06	.96	.57 (1)
*Dependent variable*: *Gap-filling Errors*				
Differential valence	.08	.10	1.09	.64 (1)
Differential arousal	- .15	.07	.86*	4.68* (1)
Differential valence x Differential arousal	.05	.06	1.05	.62 (1)
*Dependent variable*: *Hits*				
Differential valence	.01	.08	1.01	.01 (1)
Differential arousal	.12	.05	1.13*	5.13* (1)
Differential valence x Differential arousal	.03	.05	1.03	.44 (1)

Random effects were Participants and Scripts. Number of observations was 2436 for Hits, 1764 for Gap-filling Errors, 600 for Causal Errors. Number of participants = 75. Number of scripts = 8.

*p < .05

***p < .001

For gap-filling errors, differential valence did not have a significant effect, *χ*^*2*^(1) = 0.64, *p* = .42 (model with differential valence: AIC = 1474.5; model without differential valence: AIC = 1473.5). A significant main effect of differential arousal emerged, *χ*^*2*^(1) = 4.68, *p* = .03 (model with differential arousal: AIC = 1470.4; model without differential arousal: AIC = 1473.5); as shown by the parameters reported in Table 4, this indicates decreasing probability of gap-filling errors with increasing arousal ratings. No interaction between differential arousal and valence emerged, *χ*^*2*^(1) = .62, *p* = .43 (model with the interaction: AIC = 1473.6; model without interaction: AIC = 1472.2). The relative likelihood of the model with differential arousal as the predictor with respect to the corresponding model with group was 19.1, again indicating clear evidence in favour of differential arousal.

Finally, also for hits, no main effect of differential valence was found, *χ*^*2*^(1) = .01, *p* = .94 (model with differential valence: AIC = 2149.2; model without differential valence: AIC = 2147.2), but significant main effect of differential arousal was found, *χ*^*2*^(1) = 5.13, *p* = .02 (model with differential arousal: AIC = 2144.0; model without differential arousal: AIC = 2147.2); as shown by the parameters reported in Table 4, this indicates increasing probability of hits with increasing arousal ratings. No interaction between differential arousal and valence emerged, *χ*^*2*^(1) = .44, *p* = .51 (model with the interaction: AIC = 2147.3; model without interaction: AIC = 2145.8). The relative likelihood of the model with differential arousal as the predictor with respect to the corresponding model with group was 0.70, this time indicating that while both group and differential arousal are good predictors of hits, it is not possible to establish which of the two is a better predictor.

## Discussion

Mood was found to affect true and false memories for events when induced before retrieval. Specifically, arousal—but not valence—predicted the probability of committing memory errors: both negative and positive mood groups (similar for arousal levels) were less likely to produce causal errors compared to the neutral group. This is even more evident when we directly used the difference in the participants’ ratings between pre- and post-mood induction as the predictor of memory errors. Indeed, the increase in arousal after exposure to emotional scenes (compared to the individual’s baseline) predicted the extent to which individuals would be later influenced in their memory performance. Larger increases in perceived arousal predicted lower probability to produce overall false memories (i.e., individuals were less likely to incorporate unseen events into their memories), and higher probability to correctly recognizing target events. On the contrary, differential valence did not predict memory performance. Most important, analyses of the relative likelihood of the models clearly indicated that self assessed mood ratings predict false memory performance better than mood groups. This is important because it shows that assigning participants to different mood groups and subsequently conducting between-group comparisons–while still useful–may not be the most adequate and powerful way to test the effect of emotional states on memory; for example, in our study, a significant effect of mood on gap-filling errors emerged only when we analyzed arousal as a continuum. Analyses on the subjective ratings allow to evaluate the individual’s perception of their changing emotional state, and to take into consideration individual differences in the extent to which participants respond to mood induction (and subsequently how this impacts their memory performance).

The present findings extend previous evidence that arousal, in some circumstances, reduces the susceptibility to false memory (e.g., [8]). In their study, English and Nielson found that arousal exerted its influence on consolidation processes (i.e., when the memory trace has to be maintained in long-term memory). While we cannot rule out that consolidation processes are still at play in our study, we argue that arousal affects retrieval to a higher extent. Participants were not instructed to remember the photographs presented at encoding (i.e., the subsequent memory test was incidental) and, furthermore, they performed filler tasks during the retention interval that distracted them from thinking of the viewed photographs. Finally, participants were exposed to mood-induction immediately before they had to retrieve the episodes. All these aspects suggest that the current findings are due to processes taking place at retrieval. Future research should address whether mood affects false memory to a different extent depending on the memory stage involved (encoding, consolidation or retrieval). The findings relative to subjective experiences of recollection and familiarity–described below–sheds further light on the role of the retrieval processes involved in the current study.

Given that arousal affected both true and false memories, there may be a common mechanism that should explain the current findings. Most of the available research on the effects of arousal and stress on memory highlights the impairment of memory performance when arousal is experienced before retrieval [e.g., 12]. One recent study shows that whether memory retrieval is impaired depends on time, and on the specific physiological processes involved [35]. These authors tested the hypothesis that if individuals are experiencing stress and are simultaneously required to retrieve previously studied information, then an immediate release of adrenaline and noradrenaline from the autonomic nervous system occurs and could facilitate memory retrieval. Indeed, noradrenergic arousal has been linked to successful memory retrieval [36]. However, if retrieval takes place several minutes after the stressful experience, then the hypothalamus-pituitary-adrenal axis triggers a cascade of events which lead to the secretion of glucocorticoids (i.e., cortisol) and would impair memory retrieval. They found that, while a specific effect of stress on memory was not found, individual differences in change in blood pressure (indexing activity of the autonomic nervous system) were positively correlated with memory retrieval when participants were still under stress (but not 25 min after stress). Conversely, individual differences in cortisol response were negatively correlated with memory retrieval 25 min post stress (but not when participants were under stress). Schönfeld and colleague [35] argue that, consistently with previous research, rapidly occurring noradrenergic mediators improve the capacity of the hippocampus and the prefrontal cortex, while cortisol is not yet at its peak. Given that our retrieval task took place immediately after mood-induction (and thus individuals were under the effects of arousal while performing the retrieval task), we speculate that our findings may be consistent with this view that arousal enhances memory when experienced during retrieval. Furthermore, our findings are consistent with the U-inverted function relation between arousal and level of performance, described by the Yerkes-Dodson law [37] (where performance increases with increasing levels of arousal only up to a point, after which performance drops), assuming that the arousal level induced in the current study was moderate-high, and the task complexity was not high.

High arousal may be associated with increased monitoring abilities at retrieval (see [38] for a review on theories on monitoring processes in false memory research). When a person is in a transient negative or positive mood, he or she may be prone to actively control the available memory traces in order to discern events that were actually experienced from those only inferred. For example, viewing the photograph of a child with pieces of a broken bottle on the floor (effect) leads to the inference that the child knocked over the bottle from the table (cause); however, when the causal scene is available at recognition, people in arousing moods tend to reject it, likely recognizing that the cause was only inferred.

The present findings on the subjective experiences accompanying false memories shed more light on the processes involved. Once false memories of causal scenes occur in individuals in negative and positive moods, they are not characterized by subjectively compelling memory traces; negative and positive moods lead to a lower recollection for unstudied events than neutral mood. However, remember responses associated with hits were not different according to the mood state, suggesting that the impact of arousal on memory performance may be stronger for memory distortions than memory accuracy, at least at the subjective level.

One of the qualifying features of recollection is the ability to retrieve qualitative aspects of the studied events’ context, along with thoughts that came to mind when the rememberer first encountered the event [39]. Illusory recollection, or phantom recollection, occurs when individuals erroneously claim to remember non-experienced events, and these events are accompanied by vivid details (e.g., [40]). For example, in the DRM paradigm, when a related lure is presented at retrieval, details originally linked to the presentation of target words are recollected and mistakenly attributed to the critical lure in a process that can be thought of as “content borrowing” [41]. In the current study, it is likely that fewer contextual and qualitative details of unstudied items are available at retrieval for people in arousing mood states, reducing the risk of illusory memory. In turn, this may serve as evidence of recollection-based monitoring processing that limits the overall rate of false memories. From a dual-process perspective, the fuzzy-trace theory [40] states that under certain conditions, the retrieval of verbatim traces (i.e., surface features of studied events) may limit the influence of gist traces (i.e., the general meaning of studied events, which is thought to be at the base of false-memory formation). Thus, when a person is exposed to non-presented causal scenes, he or she may retrieve the corresponding effect scenes in detail and consequently reject the cause because it seems familiar but it is not recollected or felt as “encountered before.” We found evidence of a reduced subjective recollection for false memories when individuals were exposed to mood before retrieving the episodes, suggesting that mood influences retrieval processing via recollection-driven mechanisms.

The current findings may have important applications. First, not only is it important to consider the affective state of the rememberer at the time when he or she experienced some (emotional or neutral) event, it is also vital to assess the transitory affective state that accompanies the person at the time of retrieving those events from memory. People in negative and positive highly arousing moods discriminate to a higher extent between experienced and non-experienced events than people in a neutral mood and are more able to ward off false remembrance, especially that which stems from causal inferential thinking. Finally, the participants’ subjective ratings about their perceived arousal states are valuable predictors of false memory performance. To conclude, high arousal at retrieval seems a valuable predictor of diminished false memory production and enhanced memory performance.
